# Cajal and his love for Nature: a sentimental essence in the legacy of neurosciences

**DOI:** 10.3389/fnana.2024.1408783

**Published:** 2024-07-18

**Authors:** Eduardo Garrido

**Affiliations:** Hypobaria and Biomedical Physiology Unit, Department of Physiological Sciences, University of Barcelona-Bellvitge Campus, Hospitalet de Llobregat, Barcelona, Spain

**Keywords:** nervous system, neurohistology, neurosciences, environment, landscaping, scientific romanticism, nature, Santiago Ramón y Cajal

## Abstract

Santiago Ramón y Cajal (1852–1934) revolutionized the branches of neuroscience in a forceful way, and he did it with extreme delicacy and candor. His scientific writings and drawings are full of allusions to Nature, a fact that demonstrates how he saw, understood and enjoyed it with exquisite sensitivity and pressing emotion. Neuroscience awakened in him the utmost curiosity to delve into the powerful mysteries of the mind, and neurohistology allowed him to satisfy his deepest concerns for fascinating scenarios, a desire not sufficiently fulfilled throughout the fields, mountains and forests of his childhood and youth. Through that wonderful microscopic world Cajal changed the size of the dreamed landscapes but not the dimension of the longed-for adventures. Exploring and entering unknown paths he unraveled some of the greatest enigmas that the nervous system hid, but he would do so with a deep feeling toward the infinite beauty that Nature itself offered him. In short, Nature was the vital axis of Cajal’s overwhelming and complex personality, his most genuine essence and the inexhaustible source of inspiration where he poured his imagination and fantasy. He became a vocational adventurer, an insatiable explorer, a talented artist and an exquisite humanist. An eminently romantic soul who knew how to link Nature and Neuroscience with unconditional and perpetual emotionality.

## Introduction

Many prominent scientists have felt a great devotion to Nature, and the way they have understood the universe that surrounded them has been a decisive factor in their exceptional contributions to Humanity. Like other notable contemporary figures in the branches of knowledge, Santiago Ramón y Cajal can be framed under a way of intuiting science that was promulgated by the so-called ‘Naturphilosophie’ (Lewy, 1987). This source of romantic idealism, which is rooted in the pre-Socratic tradition of Ionian philosophy, reached its maximum splendor in the 19th century and had a great influence on the thought and work of many prominent philosophers, writers, artists and scientists. In its biological dimension this current was characterized by admitting Nature in a global organic conception, by conceiving the world as a projection of the observer, and by searching for and analyzing the hidden relationships between natural elements (Verweyen, 2018). A clear example was Alexander von Humboldt, considered by some to be the ‘father of ecology’, whose beautiful descriptions of the natural world impressed and inspired the young Darwin as well as many other aspiring explorers and scientists of the time.

Nevertheless, this unified view of Nature was later replaced by the rise and expansion of empiricism and experimental biology, of which Cajal was in many ways an appropriate representative. How the neuroscientist Cajal was able to reconcile both antagonistic philosophical orientations is an intriguing fact, but he probably limited his adherence to ‘romanticism’ simply because of his precocious love and reverence for Nature from his earliest childhood and his innate tendency toward some forays into the sphere of the supernatural and mysticism. Early on, Cajal observed, lived and tried to understand the natural world and its hidden intricacies with an unprecedented passion. His subsequent holistic worldview was demonstrated by a keen interest in fields as far-flung as astrology *“I indulged myself during the long winter nights in consulting the celestial charts and determining the position of the constellations”* (Ramón y Cajal, 1901, p. 62), to his dedication to insect ethology *“my observations of the life of ants* […] *my explorations of the wanderings and habits of wasps, bees, bumblebees and butterflies”* (Ramón y Cajal, 1934, p. 225). Possessed by this deep emotionality, he also showed an early artistic talent, cultivating with true devotion literature, drawing, painting and photography (Steininger and Codina-Canet, 2018), disciplines through which he tried to reproduce with the delicacy of his extraordinary skills the natural environments that made him enjoy immensely.

Such a versatile personality as Cajal had, together with his thirst for adventure and fed by fantastic readings during his youth, provoked in him a perception of existentialism that would lead him to yearn for a free and exciting life through dreamlike scenarios, but through Science he finally found the best way to fulfill the joy of venturing into Nature. By exploring and drawing tiny and fascinating landscapes of the nervous system, he managed to materialize a complete scientific dedication that he carried out with exquisite romanticism (DeFelipe et al., 2014). ‘Romantic Science’ has been characterized, among other aspects, in sensory experience, the search for mystery and wonder, and the relationship between Science and Philosophy (Hadzigeorgiuo and Schulz, 2014), traits that perhaps are experienced to their maximum degree in solitude, and that also in the case of Cajal this isolated path was traveled with an extreme quixotic purity (Williams, 1954). This strong and constant emotionality toward Nature that he possessed was the embryo from which, over time, all his disciples were imbued and nourished. A great team of people who, under the auspices of Cajal, formed the renowned Spanish neurological school (de Carlos and Pedraza, 2014; Giné et al., 2019), without which world neuroscience would not have undergone the great development it had (de Castro, 2019).

The main objective of this review is to address this very transcendental facet of Cajal’s life, where his deep thoughts, imaginative theories and valuable meditations that he developed over the years seem based on his unbridled love for Nature, and how all of this had a decisive influence and was reflected in his scientific work. Through a hermeneutical analysis and exposing a succinct sample of revealing images, as well as a careful selection of testimonies and feelings about Nature –some of them very unknown or forgotten–, the aim is to justify how he interiorized it in the depths of his soul, making Nature the fundamental axis of his vital existence.

### Nature in the genesis of the scientific artist Cajal

To understand the influence that Nature had on Cajal’s work we must go back to his earliest years of childhood, moments in which the exquisite sensitivity that characterized him could already be glimpsed, and when his strong personality began to be forged. Coming from a rural lineage and having grown up in the countryside and, therefore, being sensitively imbued with the infinite manifestations offered by Nature, was what made it easier for him to awaken his curiosity for all that was beautiful and enigmatic. During his old age he would describe the initial moments of his life in a revealing way: *“… the austere decoration with which nature wounded my virgin retina and loosened my brain”* (Ramón y Cajal, 1923, p. 12).

In those distant years, this child born and raised at the foot of the Pyrenees mountain range showed himself to be possessed of an unprecedented emotionality and an indomitable seduction for the natural scenery that surrounded him. From the beginning, Nature was an inspiring force, consolation and refuge for him, an inexhaustible source of possibilities. Let us see just a few snippets of his testimonies, of those experiences of childhood and youth lived in towns, villages and hamlets scattered throughout the remote countryside of Northern Aragon (Spain), which he himself would remember so much over the decades with great nostalgia and tenderness:

*“The beauty,* *variety and originality of the natural works attracted me with irresistible power”* (Ramón y Cajal, 1901, p. 47); *“I could not get enough of contemplating the splendors of the sun, the magic of twilight, the alternatives of plant life with its lavish spring festivals, the mystery of the resurrection of insects and the varied and picturesque decoration of the mountains”* (Ramón y Cajal, 1923, p. 17).

The sensations of freedom through physical exercise in the middle of Nature, his innate exploratory avidity and an unlimited curiosity for the unknown (Garrido, 2014) always accompanied him from the moment he began to track and carefully contemplate those wild and captivating landscapes:

*“…exploring ravines, torrents, fountains, rocks and hills…”* (Ramón y Cajal, 1901, p. 47); *“Among my irrepressible tendencies, there was a certain bizarre fondness for finding out the course of rivers and surprising their tributaries and springs”* (Ramón y Cajal, 1923, p. 103).

Likewise, his precocious talent for drawing and his early love for painting everything he admired led him to develop his own pictorial collection where he gave free rein to his enamored mind; that’s how he remembered it:

*“The pages of my album, converted into an archive and reliquary of my loves, were filled with groups of trees, bouquets of wild flowers, streams that slid over pebbles and among reeds, brambles and fields of flowers, where the nightingale and the goldfinch nested, and dragonflies and butterflies perched”* (Ramón y Cajal, 1901, p. 161).

**Figure 1:**
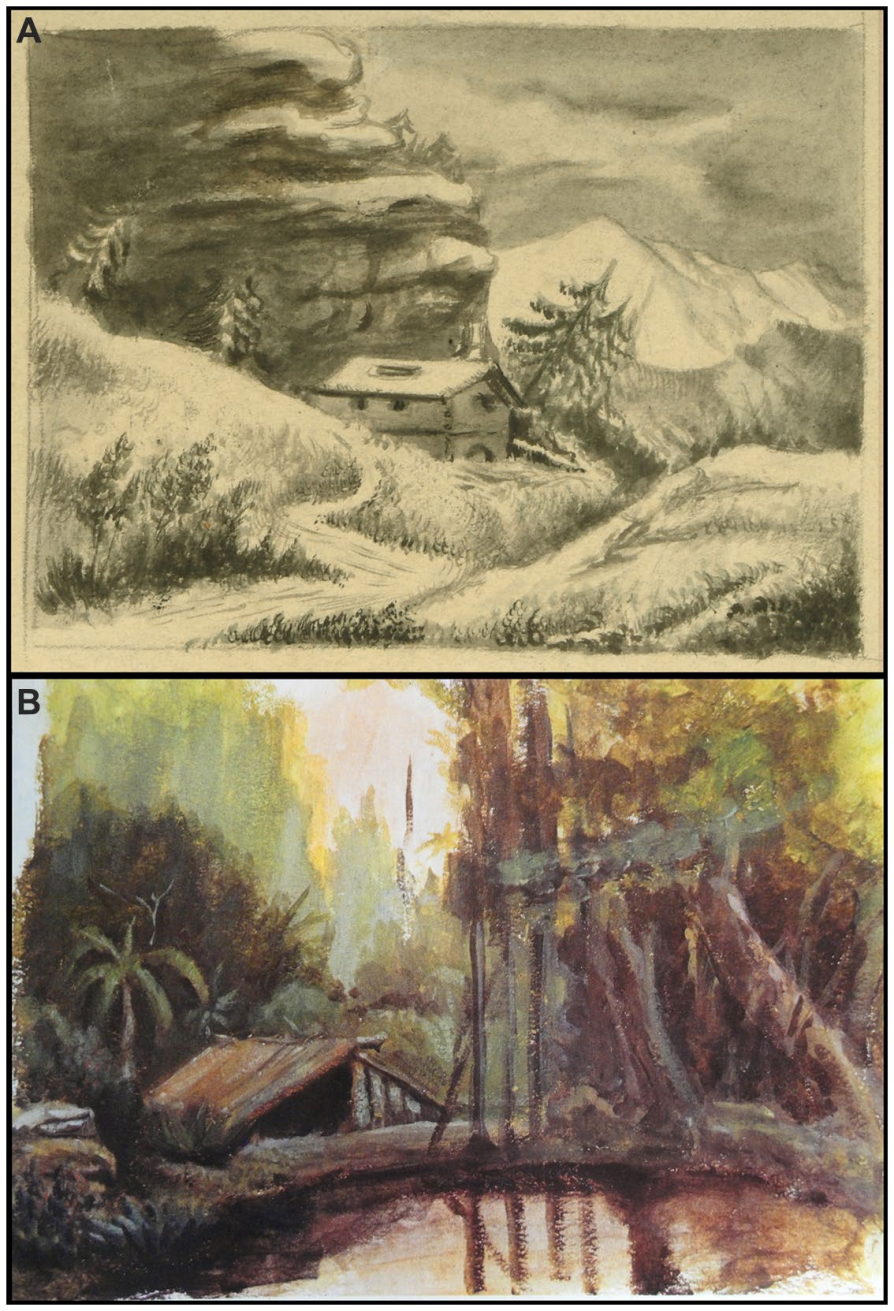
**(A)** Winter mountainous landscape painted by Cajal during his childhood (ca. 1864, Chinese ink wash in three colors). **(B)** Tropical landscape painted by Cajal during his youth (ca. 1874, oil on paper) [from Cajal Legacy-CSIC, Madrid, Spain].

Somewhat later, during his adolescence and youth, he made beautiful charcoal drawings, watercolors and oil paintings, copying the landscapes of his native land directly from Nature (Figure 1). He even painted fictitious scenes where natural environments took on special prominence: *“… to bathe my spirit in the waves of free nature; that is, to reinforce my memories with the vision of the cheerful mountains and smiling countryside of the place, objective elements of my pictorial imaginations…”* (Ramón y Cajal, 1901, p. 173). Some of his close colleagues knew how to reflect very well that energetic personality and exacerbated emotionality that deeply rooted in the young Cajal, in his fanciful temperament with almost dreamlike and uncontrollable tendencies toward the natural world, of the man who could have been and was not:

*“… his own inflamed fantasy during his long and solitary country excursions would have turned the wandering and self-absorbed young man into an incorrigible dreamer, and perhaps a visionary, if he had not, fortunately, had two innate moderating elements: interest in natural phenomena and the love of executing works with their hands”* (Olóriz, 1907, pp. 62–64).

Around 1868, finally encouraged by his father toward science, he began his medical studies in the city of Zaragoza and went to collect bones in a cemetery to reconstruct and draw them. Cajal remembered that “macabre looting” –as he defined– with words that are very revealing of an eminently imaginative and adventurous personality:

*“In the dim light of the night luminary, those skulls half buried in the gravel, and over which irreverent thistles and nettles climbed, seemed to me something like the frame of a shipwrecked ship stranded on an enemy beach”* (Ramón y Cajal, 1901, p. 264); *“My pencil took pleasure in animating the inert shells of the organism, drawing in outline the muscles that agitated them and the veins and arteries that nourished them. Adorned with the showy sail, the frame of the human ship appeared more beautiful and understandable”* (*ibid.*, p. 270).

As we see, Cajal recreates these events in a whimsical and intriguing way and reveals to us, once again, his artistic impulses. He studies with illusory eyes, seeing something more than simple morphological features in the anatomical pieces, since he recognized as a “geophysical accident” those capricious shapes that Nature had meticulously chiseled into the hardest structures of the organism. Anatomy, more than a subject, seemed to constitute an exciting landscape journey through the small details that the human body offered to him, since for an absolutely creative mind it seemed necessary to envision authentic valleys, streams and cliffs where there were none:

*“Through there –I said to myself, pointing to a bone in the skull– runs a channel where an artery ran away that pulsated, no doubt, under the impulse of passion, widening the rocky bed with its waves and shudders; beyond I see a conduit, a kind of narrow gorge opened in the living rock to shelter a nerve that seeks protection in the bone* […]*; in that other place, a ridge or protuberance appears, a kind of mountain range from which the muscle starts, that red and complicated machine where heat is converted into mechanical force, and which, in its birth and mode of activity, reminds, respectively, the river sprouting from the mountain…”* (Ramón y Cajal, 1901, p. 269).

While attending medical school, Cajal also increased his literary enthusiasm initiated during his childhood and adolescence. Most of his gripping readings corresponded to romantic authors, such as Espronceda, Zorrilla, Goethe, Hugo, Byron, Chateaubriand or Lamartine, and adventure books such as those by Verne, Twain, Dafoe, Cook, Dumas or Cervantes, as well as idealistic philosophers such as Berkeley, Fichte or Kant. All of them captivated and deeply nourished Cajal’s fanciful spirit and a certain influence is distilled in the literary style he later used in creating his own books of memoirs, essays or fiction, as well as in narrating the amazing descriptions throughout his scientific texts. But, at that time, shortly after finishing his medical studies at the age of 21, and strongly marked by the worldview of these writers, the young doctor Cajal tried to follow his innate impulse as an adventurous traveler. This is an example of how he recalled his vehement exploratory desires for beautiful imagined landscapes:

*“… I’m disgusted with the monotony and the routine of vulgar life. I feel an insatiable thirst for freedom and new emotions. My ideal is America, and particularly ‘tropical America’ […] Only there does nature appear as grandiose as it is* *varied, and the plants, eternally green, reach the height of their vegetative potential”* (Ramón y Cajal, 1901, pp. 330–331).

But he soon saw his longing to wander through idyllic dream scenarios cut short, because after becoming seriously ill during his participation in the war in Cuba, the tropical infections he contracted there and a sudden lung ailment brought him close to death. The slow recovery occurred in his childhood land and mountains –*“… bathing my soul in the heart of Nature”* (Ramón y Cajal, 1923, p. 103)– although he always carried the consequences of those illnesses that deteriorated his health and, therefore, his stubborn desires were truncated, but he would remain faithful, however, to his spirit until the end of his days. From then on, he will vary the size of the landscapes, but not the dimension of his adventures, since Science was the ideal substitute for such painful and early renunciation. Cajal tried, therefore, to appease his sadness and sorrow by continuing with a life brimming of curiosity from his laboratory table, and his great objective would be the exploration of Nature through his microscope. Soon the cellular jungles of the brain would become new and hidden paths along which to venture and pour out all the inquisitive and artistic soul of his childhood: *“… a man’s entire future is in his infancy…”* (Ramón y Cajal, 1901, p. 69).

### Nature in Cajalian neurohistology

From his solitary beginnings in the field of research Cajal could not conceive of carrying out his dedication without a dose of absolute passion and evocative enthusiasm, and perhaps this was the key to the great achievements of his neuroscientific career (DeFelipe et al., 2014). How much sensitivity their own testimonies distill, through which, with genuine and profound lyricism, the scientific Cajal is revealed to us:

*“¿What, in short, is science if not deep, clairvoyant, infinitely ambitious poetry?* […] *But, unlike the poet of appearances, the scientific poet does not sing admiring and emphatic stanzas to the murmuring brook or to the pale sparkles of Diana; He wants to drown himself once and for all in the unfathomable sea of beauty and be dazzled until blinding in the powerful luminary of truth. Heroic lover of cosmic energy, he prefers to burn like Empedocles in the volcano of light, instead of contemplating its gray smoke and haggard reflections from afar”* (Ramón y Cajal, 1905a, p. 128).

In the long moments of solitude spent in his various laboratories, especially in the stages when he carried out his experiments in his own homes –whether in the cities of Zaragoza, Valencia, Barcelona or Madrid–, Cajal displayed all the imagination and audacity through the microscopic visions. His unbridled precocious love for Nature had led him to establish a close and indestructible bond with it (Garrido, 2016), turning it into fertile ground in which to clear up some of the most hidden and intriguing enigmas that it reserved:

*“Nature is hostile to us because we do not know it: its cruelties represent revenge against our indifference. Listening to its intimate heartbeats with the fervor of passionate curiosity is equivalent to deciphering its secrets.* […] *In what more noble and humanitarian enterprise can intelligence be used?”* (Ramón y Cajal, 1917, pp. 581–582).

First, he delved into the dense forests of the brains of the less complex species on a phylogenetic scale, and then he assaulted those of human beings. As a daring naturalist, and guided by a compelling fervor, he told us with assertiveness in his memoirs:

*“… and I embarked on the undertaking confident that in that dark jungle, where so many explorers had been lost, I would be allowed to catch, if not tigers and lions, then some modest game disdained by the great hunters”* (Ramón y Cajal, 1923, p. 242); *“Microscope at the ready, I launched, then, with my usual ardor to the conquest of the alleged anatomical characteristic of the king of Creation, to the revelation of those enigmatic, strictly human neurons, on which our zoological superiority is based”* (*ibid.*, p. 300).

As is well known, Cajal studied in exquisite detail not only the histology of the human nervous system, but also that of other vertebrates, including species of the animal world such as primates, bovids, equids, felids, canids, as well as different types of birds, fish, rodents, reptiles, and batrachians; he also did not forget to study certain genera of invertebrates, such as mollusks, annelids, and arthropods (García-López et al., 2010). Confident in elucidating differential qualitative phenomena, he set out to simplify the most confusing cellular forests by studying early phases of ontogeny, putting into it once again all his intuition, curiosity and aesthetic sense, subjugating at all costs any obstacle that might arise in the pristine landscapes through which he ventured to explore:

*“… It was about inquiring how the roots and branches of those trees end in the gray substance, of that jungle so dense that, due to its refinement of complication, it lacks voids, so that the trunks, branches and leaves touch everywhere.* […] *fearlessly exploring the adult jungle, clearing the land of bushes and parasitic plants, and isolating, in short, each arboreal species* […]*. Since the adult forest is impenetrable and indefinable, why not resort to the study of the young forest, as it were, in its* *vivarium state?”* (Ramón y Cajal, 1923, p. 200).

This brilliant intuition that Cajal possessed led him to explore the brain in its simplest form of development, that is, in its embryonic state, and thanks to his knowledge of chemistry, which he tested with tenacity in some histological techniques, he obtained staining variants that allowed him to observe any nervous structure with reliability. To this end, Cajal perfected the silver nitrate staining method –discovered by Camillo Golgi (1843–1926)– by using a double metallic impregnation and modified the induration time and the concentration of the osmium-bichromic solution. Years later he also introduced the uranium-nitrate and the gold-sublimated impregnation techniques. All this new methodological approach to the nervous system led him to obtain excellent results that were constantly reproducible, a fact that would allow the vast majority of researchers of the time to follow in his footsteps. The studies he undertook were consummated over the years in a plethora of sublime discoveries that led to the ratification of his famous revolutionary postulates on the nervous system: cell independence, the direction of the electrical impulse, chemotactism guiding the growth of appendages, the establishment of new intercellular contacts and even the regeneration of fibers injured or degraded. But, despite the complexity of it all, the leisurely reading of his scientific texts is comforting, since Cajal knows how to accompany the reader through a captivating narrative, leaving the reader absolutely absorbed, overwhelmed. And this is not only because of the originality and abundance of his observations and discoveries, but also by the extreme meticulousness of his descriptions and the delicate beauty of the numerous drawings they contain. Given his usual careful precision, it is striking to note how he chose to resemble some of the details of his microscopic visions by means of very genuine semantics and sketches. This appears to be a simple exposition, but it is an engaging one, with an endless number of appellatives and symbolism with facts and elements present in Nature itself. Cajal’s veneration for it is constantly evident, and he uses it opportunely and in subtle and evocative way.

In the following examples, we can see that when he described the cells of the nervous system, or tissue segments and territories, on numerous occasions he used a nomenclature and expressions more typical of a botanical atlas:

*“vines,” “shrubs,” “bushes,” “bramble,” “weeds,” “petals,” “leaves,” “trunk,” “twigs,” “rootlets,” “vegetation,” “fronds,” “tuft,” “pear,” “onion,” “olive grove,” “fields of ears,” “hyacinth hedges,” “pinkish efflorescences,” “flower pot,” “pine branches,” “tops in the forest,” “impenetrable jungle,” “virgin manigua,” “creeper arborization,” “beds of ivy that branch a wall,” “mossy tree coverings,” “acacias surrounding the gardens,” “lianas along the branches of a tropical tree,” “degenerate and die like rotten fruit fallen from the tree”*…

He even used terminology and locutions concerning entomology and zoology:

*“swarm,” “hive,” “spider,” “sponge,” “wrinkles and humps,” “bristling with thorns,” “penniform tail,” “robust foot,” “giant’s arms,” “horse tail,” “tentacles of an octopus,” “foot of palmipeds,” “antlers of a deer”* …

To describe some of the phenomena he observed in his histological preparations, Cajal also used analogies with cosmic and environmental aspects, as well as the use of various action verbs and curious qualifying adjectives that denote before his eyes the presence of a living and pulsating Nature:

*“nebula,” “infinity,” “starry,” “comet,” “half-moon,” “labyrinthine,” “escorted,” “stragglers,” “wandering,” “ferruginous,” “monstrous,” “docile,” “lazy,” “withered,” “wriggle,” “vibration,” “meandering,” “lash,” “sucking,” “explorers,” “partners,” “drill,” “drain,” “fly,” “run,” “jump”* …

He also resorts to using numerous country and mountain similes, comparisons with peasant tools, and even colloquial expressions of rural environments:

*“moor,” “sown,” “plowed,” “threshed,” “palisades,” “cavities,” “peak,” “ridges,” “avalanche,” “spouts,” “fountains,” “lagoons,” “islets,” “gulfs,” “affluents,” “bow,” “rod,” “harpoon,” “reel,” “nosebag,” “basket,” “sickle,” “staff,” “elements that populate it,” “very different localities,” “born in this region,” “border provinces,” “poor and rich lands”* …

Likewise, Cajal seemed to be surprised by the characteristics acquired by certain itineraries and places where some nerve fibers ran. In his suggestive descriptive narratives, we observe as if he were traveling with these fibers through remote settings, along mountain trails and marine or river scenery:

*“wavy path,” “at the top of the terrain,” “linking both banks,” “descending route tracing twists and turns,” “the labyrinthine and extensive nature of the journey,” “the blue in these landscapes,” “from the bridge upwards,” “cross through different landscape settings,” “commonly traveling in a zig-zag,” “route through the* *various terrains,” “walking disoriented and groping,” “pushing and overcoming the obstacles encountered,” “crossing the void,” “thrown onto the way of an evolution that will never walk again,” “disoriented and turning in on itself, like a ship that does not want to move forward and waits for the opportunity to enter port”* …

This vast, suggestive and marvelous terminological universe, chosen and delicately adapted by him, appears in isolation or repeatedly throughout his writings and scientific books, from which I have extracted the examples previously selected (Ramón y Cajal, 1891, 1893, 1899a, 1904, 1913, 1914, 1917, 1924, 1933). The fact that he chose such expressions and words might seem simple, but, as is well known, Cajal showed great perfectionism and extreme thoroughness in his work. Therefore, he also liked to be very exact in describing what he saw through the microscope and, likewise, by capturing such visual observations in fabulous micrographic drawings. Far from erring in the similarities that he established, his imaginative sphere reveals to us the marked sensitivity and emotionality of the researcher, of the scientist with a clear but suggestive aesthetic expressiveness, of a man of extreme romantic simplicity and a lover of the countryside and Nature as he always was.

In this same topic we find neurophysiological examples that are also very demonstrative, since he established allegories with typical high mountain views: *“… the currents arriving from the sensory organs would flow out, and from where the motor or centrifugal conductors would spring, like rivers emerging from alpine lakes”* (Ramón y Cajal, 1917, p. 114). In other illustrative cases Cajal even shows us a possessive love for some of his findings that reminded him of aspects of Nature: *“… between my nests, creeper or mossy fibers…”* (*ibid.*, p. 309). This burning emotionality is also glimpsed when he used very suggestive expressions when he observed that between the nerve fibers, they ‘sniff,’ ‘hug’ and ‘kiss’ each other, to define the successive action undertaken by cellular expansions in search of the final destination, establishing the most suitable dendritic contacts. Through these extraordinary similes, Cajal not only justified the magical procedure of Nature in the development of these synaptic connections, but he came to narrate this fact as if it were a true romantic fable:

*“Like the miner, who digs blindly in search of the missing stratum, the protoplasmic shoots try* *various tracks until they find the right one.* […] *Nature proceeds like the gardener who straightens and favors well-directed shoots and prunes vicious or superfluous ones.* […] *What mysterious forces preside over the appearance of expansions, promote their growth and branching, cause the congruent emigration of cells and fibers, according to predetermined directions and as if obeying a wise architectural plan, and establish, finally, those protoplasmic kisses, the ‘intercellular articulations’, which seem to constitute the final ecstasy of an epic love story? …”* (Ramón y Cajal, 1923, p. 226).

On certain occasion he recreated these phenomena of intercellular alliance as if it were a fictional scene between solitary beings called to unite by themselves or, perhaps, governed by the powerful and enigmatic impulse of a higher entity:

*“… adopting predetermined directions, and establishing without revolts or mistakes, as guided by an intelligent force* […]*. These secret attractions that precipitate with considerable speed, and despite obstacles and distances, some elements over others, constitute a particular case of the sovereign problem of ontogenic evolution and an eloquent example of the great solidarity that reigns among all the inhabitants of the organic colony”* (Ramón y Cajal, 1899a, p. 554).

We observe how in these passages Cajal’s expressiveness reaches an arrogant devotion, but, of all his neuroscientific research, the one that perhaps seduced him with the most irresistible sentimentalism was the study of the enigmatic biological matter sensitive to light: *“… the retina, the oldest and most pertinacious of my Laboratory loves”* (Ramón y Cajal, 1917, p. 289); *“… where Nature has surpassed itself is in the construction of the retina…”* (Ramón y Cajal, 1934, pp. 16–17). In various passages referring to other cerebral areas, Cajal also recalled the fruitful histological staining of those tissues that appeared invisible and the consequent beautiful findings that revealed the most intimate aspects of the brain structure. The following two testimonies show us, once again, the exquisiteness of the naturalist man:

*“… The naive admiration of the cellular form constituted one of my most gratifying pleasures. Because, even from a plastic point of view, the nervous tissue contains incomparable beauties. Is there any tree in our parks more elegant and leafier than the Purkinje corpuscle of the cerebellum or the ‘psychic cell’, that is, the famous brain pyramid?* (Ramón y Cajal, 1917, p. 156).

*“… the cellular hive is offered to us without veils; It seems that the swarm of diaphanous and invisible infusoria is transformed into a flock of painted butterflies”* (*ibid.*, p. 432).

**Figure 2:**
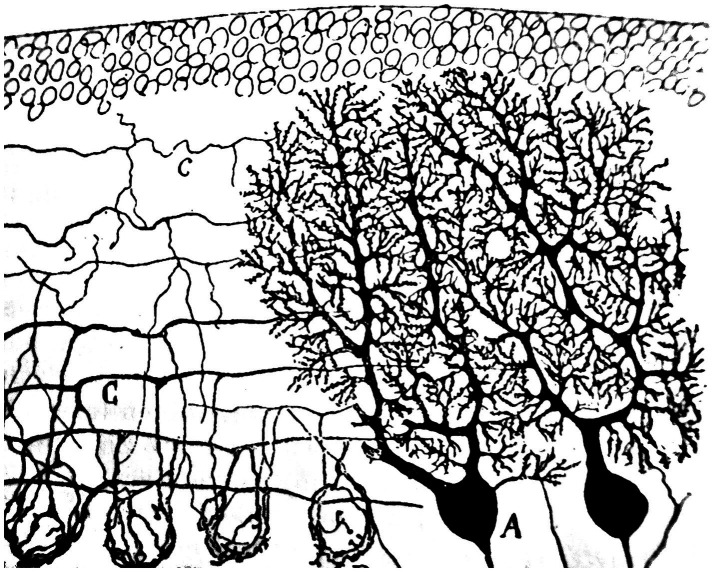
Purkinje cells, nervous baskets and stellate cell axons of a young cat [from Ramón y Cajal (1904, p. 317)]. The image resembles a countryside landscape with its bushes, groves, farm furrows and rocky walls.

**Figure 3:**
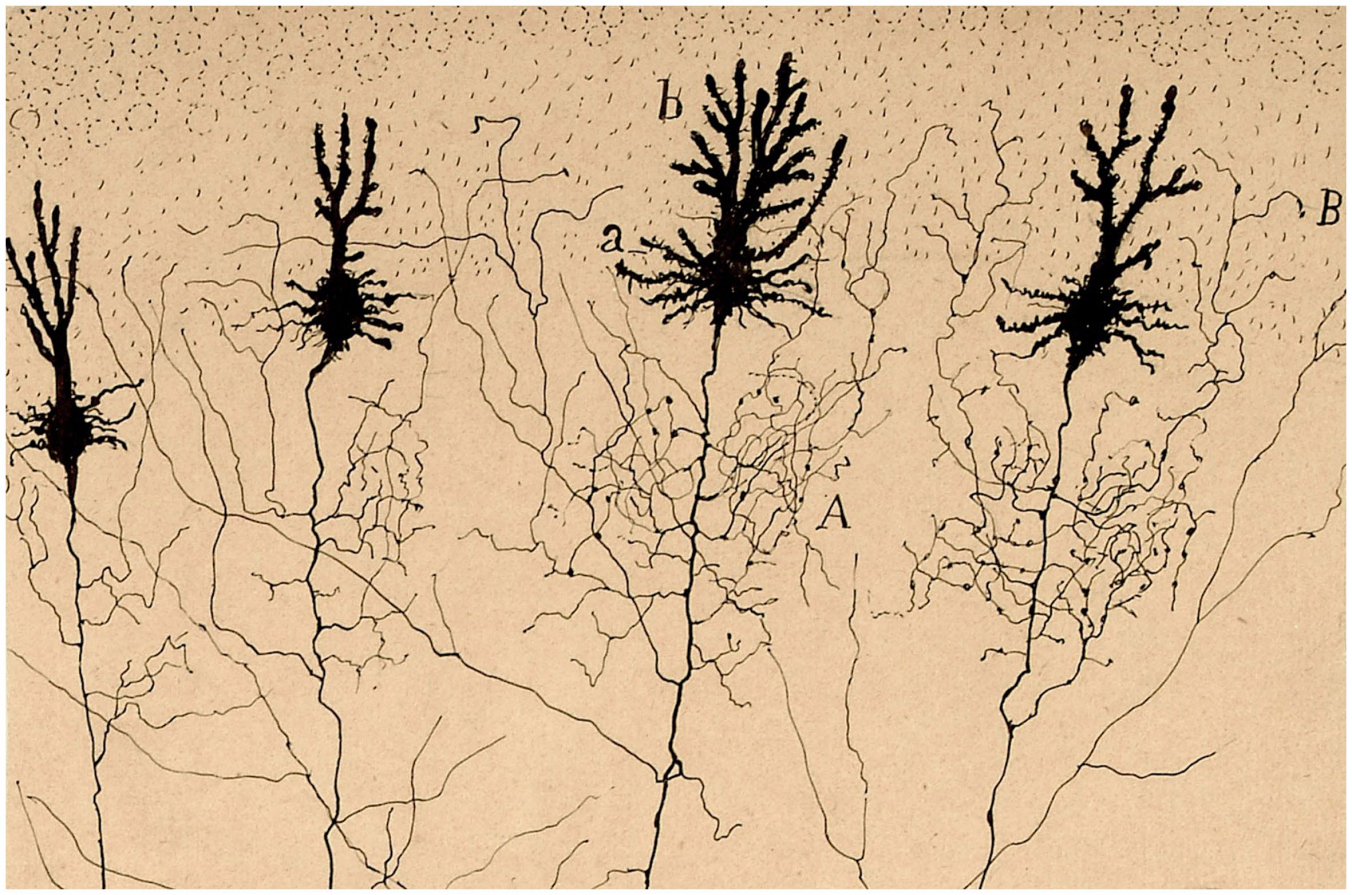
Purkinje cells of a newborn child [from Cajal Legacy-CSIC, Madrid, Spain]. The image resembles a bouquet of wildflowers.

**Figure 4:**
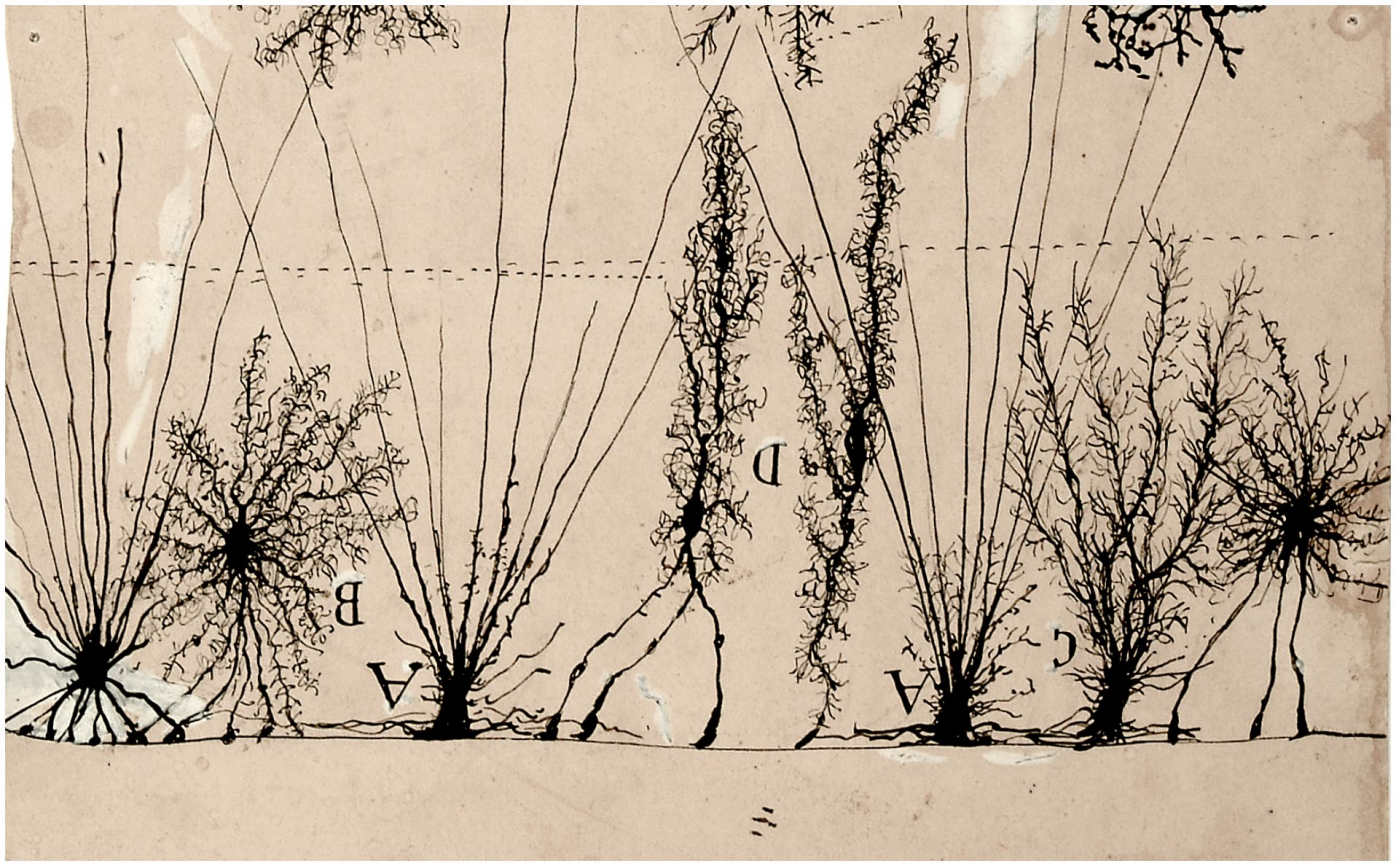
Glial cells of the superficial layer of the brain of a child (partial and inverted figure) [from Cajal Legacy-CSIC, Madrid, Spain]. The image has similarity with plant fronds, bushes and ferns.

**Figure 5:**
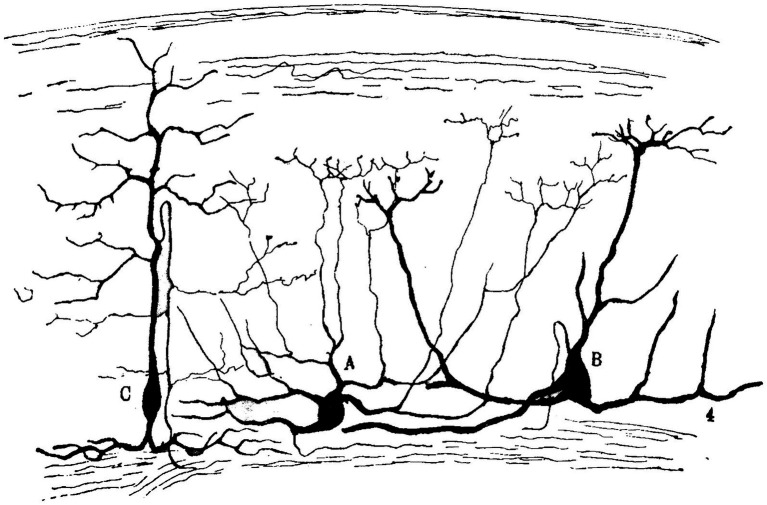
Ganglion cells and axons [from Ramón y Cajal (1904, p. 512)]. The image resembles a winter landscape with bare trunks and branches under a sunset sky.

**Figure 6:**
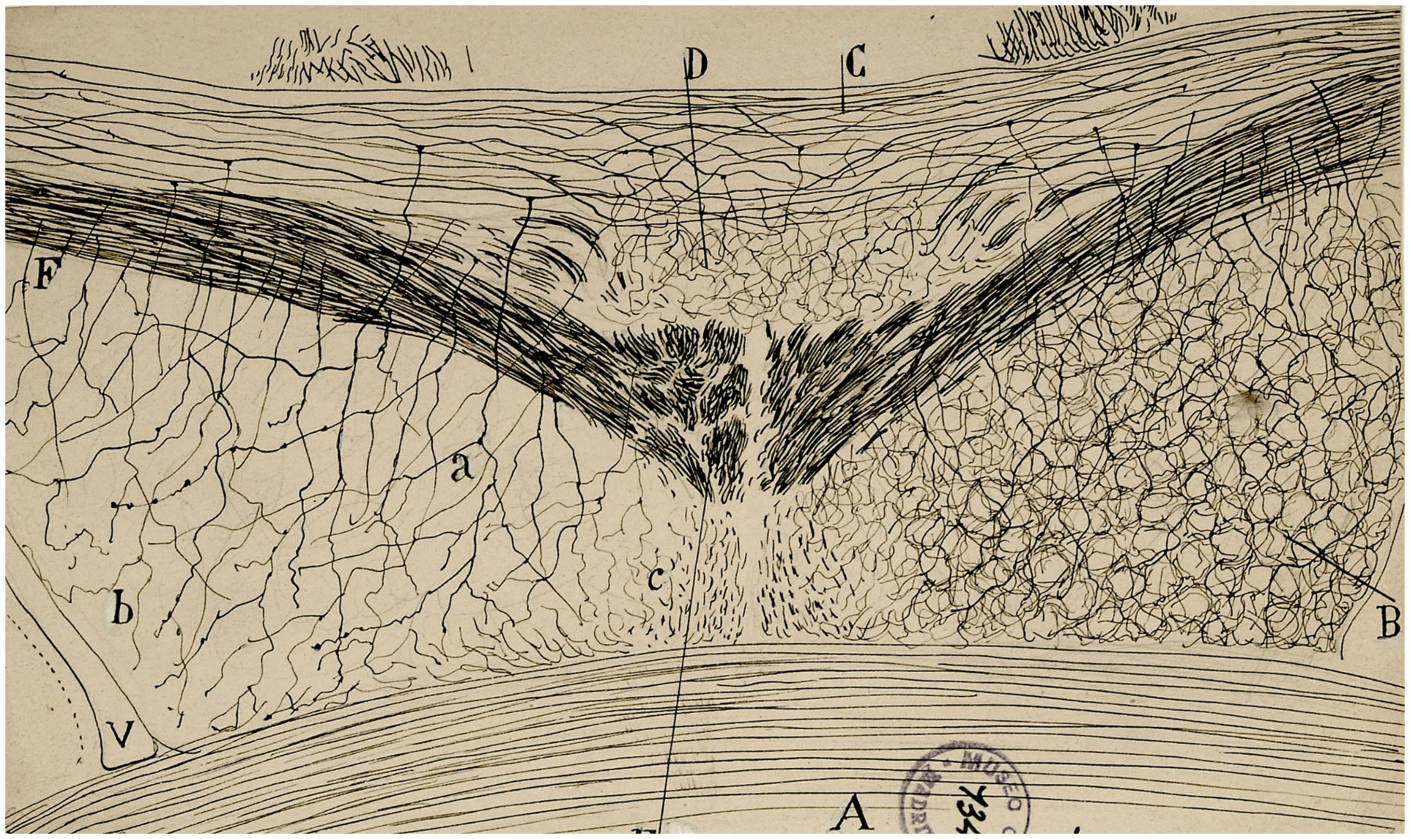
Histological section at the level of the septum of a newborn mouse [from Cajal Legacy-CSIC, Madrid, Spain]. The image resembles a meadow with some spikes swaying in the wind and a slope invaded by brambles.

**Figure 7:**
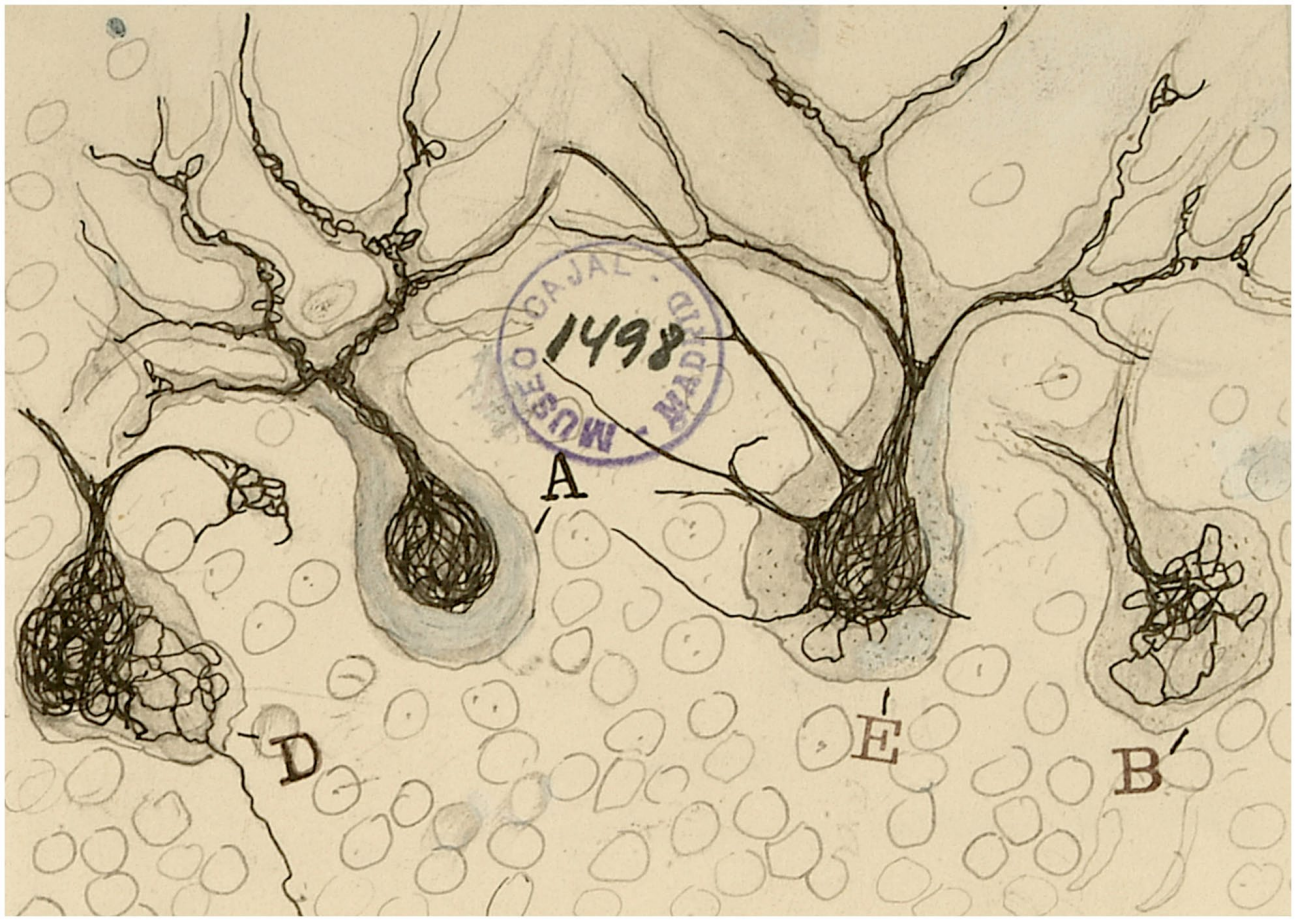
Purkinje cells from the cat cerebellum damaged by tissue compression [from Cajal Legacy-CSIC, Madrid, Spain]. The image resembles octopuses with their tentacles.

**Figure 8:**
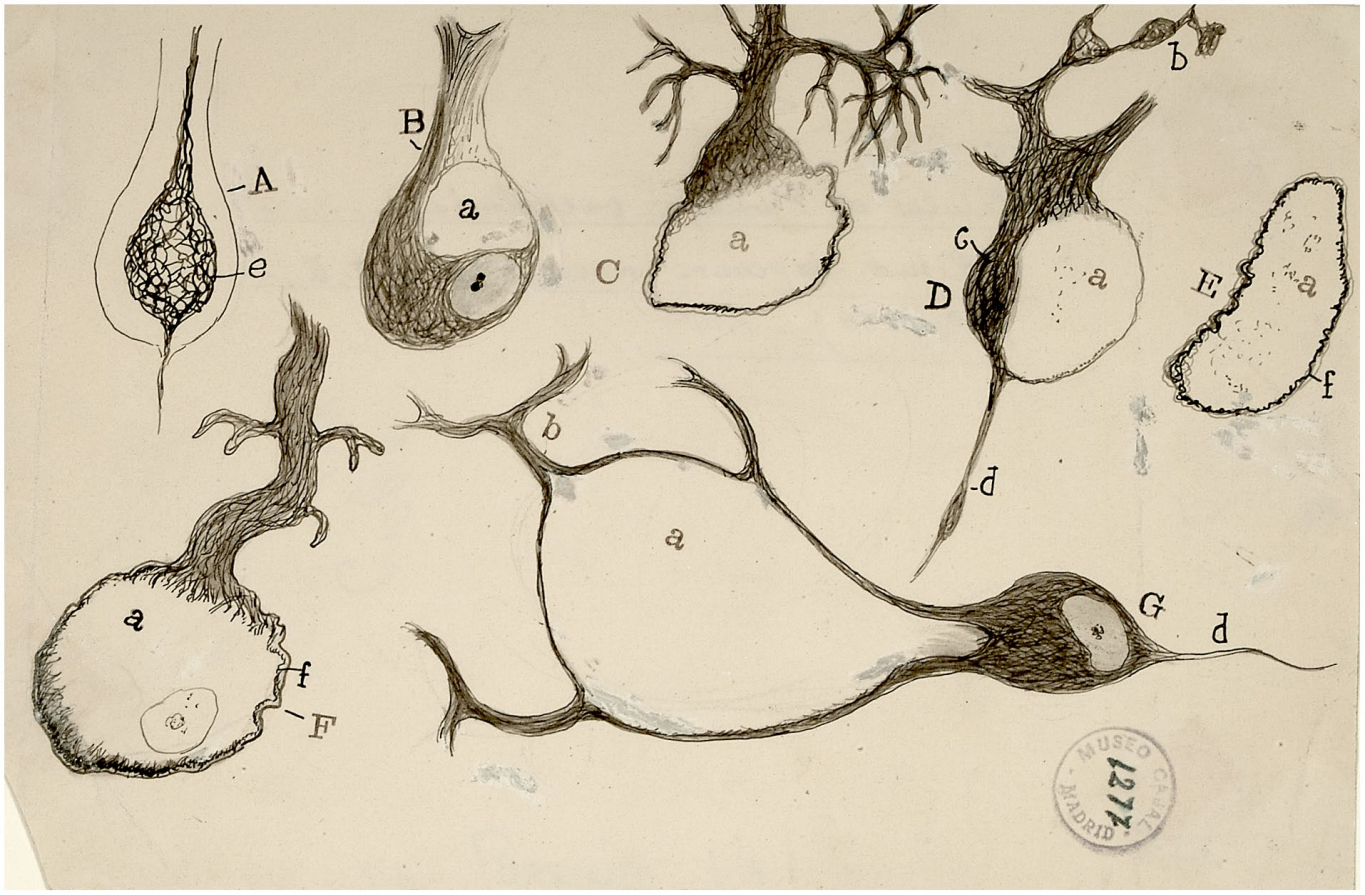
Cat Purkinje cells involuted by vacuolization [from Cajal Legacy-CSIC, Madrid, Spain]. One of the images resembles a sketch of a penguin.

The rich lexical and phraseological roots so evocative of Nature, that Cajal constantly manifests in his observations of microscopic neuroanatomy, express the perpetual ‘robinsonian’ effusion of an adolescent emotion, or perhaps could represent a longing for an unsatisfied thirst for adventure due to the early illnesses suffered in his youth (Albarracín, 1997). We can see that many of his pictorial creations of nervous tissue have close similarities with the natural world, the one that was so sealed in his mind from his earliest childhood, when he delighted in contemplating the infinite details that country life offered him. A brief sample of his neurohistological drawings have been selected here, made with very simple strokes but demonstrative of their similarity to natural countryside environments (Figures 2–8). Cajal always did an imaginative introspection before recreating with his pencils that wonderful world he observed:

*“Is it possible that the blue of the skies, the green of the meadows, the song of the nightingale and the perfume of the flowers are not in the objects, but in us? So, when I think I paint Nature, in purity I am only portraying myself, or rather, interpreting a certain agitation of my nervous cells corresponding to a certain supposed palpitation of the external ether”* (Ramón y Cajal, 1901, p. 249).

Driven by his temperament and his initial fruitful histological discoveries, Cajal wanted to play a leading role in achieving unprecedented advances in neuroscience. He showed a strong desire for glory and, by his stubborn example, to put his country on the map of world science, but behind his complex personality always loomed his emotional and imaginative side. This fact was revealed, especially, through his sketches and drawings of nervous tissue, turning many of them into a veritable explosion of art (Newman et al., 2017; DeFelipe, 2018; de Castro, 2021). The fact that he succumbed to scientific and artistic hedonism is not an arbitrary coincidence, since the fundamental feature of Cajalian sensitivity and aesthetics is that beauty, to be such, had to imitate Nature. His artistic aesthetic can range from a classicism with Platonic-Aristotelian roots to the most genuine romanticism: the love of beauty and balance. Classicist aestheticism and the aspect of science conceived as art, since Cajalian neuronal morphology could be traced back to the Goethean conception of the forms of Nature as a living organism metamorphosing with exquisiteness in an incessant movement of creation and disappearance of life. In view of the feeling of attractiveness that he experiences in natural phenomena, Cajal expresses himself as if they had been made to achieve harmony between them and our spirit. The human being, by freely producing the beauty, adds to Nature the quality of the freedom, beyond mechanical causality. As a classical scientist and positivist, Cajal thinks that Nature obeys a strict order, whose laws he seeks to reveal, but he keeps in mind the vocation for freedom and the need for voluntary action.

The rationalist and enlightened classicism is demonstrated in all his scientific texts, but emotion is constant in all his neurohistological drawings, from the neatest order and symmetry to, even, a certain apparent chaos and confusion. Many of these pictorial sheets appear to us as a complicated network of dots and lines, especially those that represent large histological sections where complex neuronal circuits are integrated between various brain structures. Other sheets present simple and diaphanous lines, but always show a seductive harmony. Any of them leaves anyone who observes them astonished, since they are of unprecedented exquisiteness, extravagant perfection and evocative seduction: *“The supreme beauty and elegant variety of the nervous grove”* (Ramón y Cajal, 1917, p. 156). Magnificent illustrations that have been compared to those of other renowned scientists, also great lovers of the natural environment and whose creative prestige was unquestionable, such as the botanist Jan van Huysum or the ornithologist John James Audubon (Williams, 1954). Even some cytological forms drawn by him remind us of surrealist art and, despite the fact that Cajal abhorred such modern pictorial trends, his neurological drawings served as inspiration for paintings by Salvador Dalí; likewise, certain histological sketches that emerged from Cajal’s hand have been compared to the unmistakable undulations and swirls of the landscape canvases painted by Vincent van Gogh (Newman et al., 2017), the nervous vitality and intensity of vision of Diego Velázquez (Jacobson, 1993), or the water deluges of Leonardo da Vinci (Saltz, 2018).

Through the thousands of sketches and pictorial recreations on the histology of the nervous system that he created throughout his life, he always based his unbridled love for the pretty and fine textures that Nature showed him through the magnifying optics. How many of those distant sleepless nights of his beginnings, absorbed in his efforts under the amber and flickering atmosphere of a lamp, did the light of a new truth dawn, of a revelation in the face of an intuitive suspicion proudly pursued. In reality, for Cajal all that recreation represented, as in his childhood, a mere game of tests and trials, but it was the concern to be the first to contemplate an enigma, to reveal a hitherto hidden secret and be able to recreate it artistically with his pencils, which justified his determined and stubborn assault toward unknown places of brain microanatomy: *“… discovering hidden islands or virginal forms that seem to wait, since the beginning of the world, for a worthy contemplator of their beauty!”* (Ramón y Cajal, 1917, pp. 156–157).

The simple fact of entering and recreating himself in such tiny landscapes added even more fascination to his adventurous endeavor. To explore new and recondite terrain and, thus, to be able to contemplate with wonder all those scenarios of abundant splendor that remained hidden within the fine texture of the organ that governs the mind, was an exquisite emotion that it seemed to have reserved only for him. Working as a solitary and humble artisan, he liked to tenaciously probe that complex organic structure, always hypnotized in his efforts as an explorer and carried away by the wildness of a candid romantic effusion: *“In Science, as in life, the fruit always comes after love”* (Ramón y Cajal, 1899b, p. 77); *“… scientific conquests are creations of will and offerings of passion”* (Ramón y Cajal, 1917, p. 102).

Absorbed by the diligent study of these cells, Cajal let himself be carried away by such emotional intensity that it was as if he were immersed in a microscopic world in action, inhabited by imaginary beings. This curious facet was magnificently recalled by the prestigious British neurophysiologist Charles Sherrington, since both met closely when Cajal was invited by the Royal Society of London to give the *Croonian Lecture* (Ramón y Cajal, 1894a). Very revealing are the vibrant words that Sherrington used to preface a book about the figure of Ramón y Cajal, published a few years after the death of the Spanish savant man:

*“A trait very noticeable in him was that in describing what the microscope showed he spoke habitually as though it were a living scene. This was perhaps the more striking because not only were his preparations all dead and fixed, but they were to appearance roughly made and rudely treated* […] *The intense anthropomorphism of his descriptions of what the preparations showed was at first startling to accept. He treated the microscopic scene as though it were alive and were inhabited by beings which felt and did and hoped and tried even as we do. It was personification of natural forces as unlimited as that of Goethe’s Faust* […] *We must, if we would enter adequately into Cajal’s thought in this field, suppose his entrance, through his microscope, into a world populated by tiny beings actuated by motives and strivings and satisfactions not very remotely different from our own.* […] *Listening to him I asked myself how far this capacity for anthropomorphizing might not contribute to his success as an investigator. I never met anyone else in whom it was so marked”* (Sherrington, 1949, pp. 13–14).

During his last weeks of life, already very weak and bedridden, Cajal continued sketching with trembling lines some neuroanatomical drawings that were great discoveries for science. In a note addressed to his friend and colleague Rafael Lorente de No (1902–1990), he sketched a drawing representing the dendritic spines discovered by him many years ago; the small and simple sketch, made with absolute delicacy just 2 days before his death, is reminiscent of the stem of a rose bush (Yuste, 2015). Science has now demonstrated how continuous stimulation can shape the microanatomy of nervous tissue by increasing the density of such cellular appendages, as well as the leafiness of dendritic branches, a fact that Cajal already intuited in his first neurohistological investigations. In an attempt to define this malleable adaptive capacity, he quickly adopted the term *“plasticity”* (Ramón y Cajal, 1894b, p. 12), as he had previously argued for the impact of continued mental exercise on cortical neuronal interconnection (DeFelipe, 2006). Although Cajal reflected in his scientific texts this potential plastic dynamism that the nervous system possessed, he also wanted to convey this fact in a more poetic way, using his usual expressive beauty in the following two advices that allegorize Nature:

*“… every man can be, if he sets his mind to it, the sculptor of his own brain, and that even the worst endowed, from a mental point of view, is capable, like poor but well-cultivated and fertilized lands, of rendering a copious harvest”* (Ramón y Cajal, 1899b, p. 9).

*“Sculpt your brain, the only treasure you have. You lack fields to cultivate and gardens in which to enjoy yourself; So, work the field of understanding and decorate and adorn the garden of fantasy”* (Ramón y Cajal, 1905b, p. 285).

The artisanal and artistic way in which Cajal had to approach neuroscience is undoubtedly far from that of scientific practices subject to current technological development, whose purpose is that the global physiological systematization of the nervous system be completed and new therapeutic strategies toward the spectrum of neurological diseases and mental disorders be addressed. This is the great challenge of contemporary neuroscience in the 21st century, a new era of international collaboration on country-led initiatives that combine a multidisciplinary approach using powerful bioengineering, neuroimaging, gene sequencing and supercomputing techniques to delve into the structure and function of all levels of brain organization, as in the ambitious ongoing ‘Human Brain’ and ‘Brain’ projects (Amunts et al., 2016; Yuste and Bargmann, 2017). Nevertheless, the new horizons to which some of these fortunate advances will lead, particularly those concerning the cognitive sphere, must necessarily be subject to legal regulations that establish strict bioethical limits, since the possibility of unraveling the ultimate mysteries hidden in the human brain or manipulating the mind and behavior, involving even the most intimate thought processes to unsuspected levels, could have unprecedented and unforeseeable social implications (Rose, 2014; Goering and Yuste, 2016).

The good end that all these neuroscientific achievements will provide will be based on the revolutionary foundations that Cajal established more than a hundred years ago with his fabulous discoveries and postulates, and his name will always be a powerful pedestal on which to raise generations of scientists. As a living and abundant spring from which emanates a passionate and pure spirit, he wanted to encourage all the continuators of his work by nourishing them with his example and his suggestive beautiful eruditions on neuroscience and Nature. In my opinion, the following two samples are manifestations of extraordinary exquisiteness, which summarize his profound sensitivity and what was, ultimately, his existential essence and throbbing dedication:

*“The summit of truth, climbed with so many efforts, which when viewed from the valley seemed like an imposing mountain, is nothing more than a tiny foothill of a greater mountain range, which can be seen, almost unapproachable, through the fog, and which attracts us with insatiable curiosity. Let us satisfy this desire to ascend, and, taking advantage of the peaceful rest provided by the contemplation of the new horizon from the newly conquered summit, let us meditate on the plan that must lead us to higher regions and more grandiose and sublime spectacles”* (Ramón y Cajal, 1897, p. 64).

*“It was a delicious intoxication, an irresistible charm.* […] *The garden of neurology offers the researcher captivating spectacles and incomparable artistic emotions. In it my aesthetic instincts finally found full satisfaction. Like the entomologist hunting for butterflies of striking hues, my attention pursued, in the orchard of gray matter, cells with delicate and elegant shapes, the mysterious ‘butterflies of the soul,’ whose beating of wings who knows if one day will clarify the secret of mental life! …”* (Ramón y Cajal, 1917, pp. 155–156).

### Nature as essence, inspiration and balm in Cajalian creative activity and exploration of the mental sphere

From his childhood, Cajal possessed a naturalistic conception of the cosmos and, due to his innate curiosity, he always exacerbated a pure and respectful notion of his own toward everything that related the human being and to his natural environment, no matter how distant that both entities were physically. Years later, like other intellectuals of the time, his thinking fit with a merely Renaissance conception of the relationship between Man, as a species, and Nature, as an environment. He always maintained a holistic vision toward it and, despite his agnosticism, did not hide a certain pantheistic acceptance of the creation of the Universe, although his faith was professed toward the principle of the spontaneous generation of the elements.

In a certain way, his whole naturalistic inclination resembled the fiery impulse of his admired Goethe –a very prominent representative of the romantic current ‘Naturphilosophie’– or that of other great naturalist thinkers so widely read by our Spanish Nobel, such as Haeckel, Wundt, Montaigne, Keyserling, Locke, Engels, Rousseau or James. In fact, Cajal was precociously disappointed by the metaphysical and idealistic doctrines of the classical philosophers, since they did not give him an answer about the binomial ‘Man-Nature,’ so ambitiously sought by him. In that early period of his life he showed, in contrast, admiration for all the materialistic thinkers who, looking at Nature itself, tried to trace and delve into the most intimate secrets contained in it. For this reason, Cajal venerated Galileo, Newton, Laplace, Lavoisier, Humboldt, Linneo, Darwin or Einstein, among other illustrious scientists, and he did so by their respective great discoveries of seemingly simple mechanisms governed by inexorable universal laws of Nature (Lewy, 1987). Of the large number of studies carried out throughout his career in favor of ‘neuronal theory,’ perhaps one of the most interesting aspects was his innovative ability to interpret structure as the result of evolutionary mechanisms, i.e., natural selection (Ferreira et al., 2014).

The 19th century, when Cajal was born and when the romantic current ‘Naturphilosophie’ had its greatest splendor, was especially rich for the science of neuropsychological fields, domains that in turn developed in a multitude of aspects that converged. From the neuroanatomy and clinical neurology to psychoanalysis, from the biology and physiology of the senses to behaviorist methodologies, all of them trying to demonstrate what is hidden beneath the consciousness. Apparently antithetical, some of scientific areas were demarcated, and even disqualified, in the name of the ‘Scientific Positivism’ in vogue, not always coinciding in their perspectives. It is at this crossroads that Cajal comes to psychological science from medicine, but his situation as a scientist makes him cling to positivism, forced to advance discoveries about the broad scenarios offered by the jungle of the nervous system at the cellular level, fact that he carries out and establishes in a cautious and firm manner. However, from this cautious step, he launches risky psychological hypotheses and conjectures, since there the findings of neuronal histology research find their ultimate meaning. Psychology is the dome of Cajalian philosophy, because in the same way that the brain is the material basis for explaining the spiritual in human behavior, scientific psychology must base the truth of knowledge by accounting for man’s relationship with the real (Ibarz, 1994).

During the periods that Cajal spent in his country house located in the orchards on the outskirts of the city of Madrid, immersed in such a healthy environment with a beautiful view of the Sierra de Guadarrama, he overcame the neurasthenic episodes that made him get sick frequently from the age of 50 onwards. Surrounded by a soothing and vivifying calm, he tried to distract himself in the garden and, among other entertainments, he undertook the study of the behavior of insects (Ramón y Cajal, 1921). However, in this reassuring dedication, he was always assailed by a troubling objective: *“beats the determination not to cease until surprising the anatomical characteristic of the instinct”* (Ramón y Cajal, 1917, p. 570). Already many years before, during his undergraduate studies, he had written a science fiction book about a terrestrial inhabitant who traveled to the planet Jupiter, where he found humanoids ten thousand times larger than human beings. The tiny visitor managed to enter through a skin gland of one of these giants and armed with an arsenal of scientific apparatus traveled on the back of a red blood cell until he reached the brain, where he would try to discover the enigma of the mind *“the secret of the vibration of thought”* (Ramón y Cajal, 1901, p. 310). Since he elaborated this story through the Cosmos, his curiosity for the enigmatic power of the mind, for the instinctive mechanisms and for the changes in its state has not stopped growing. In fact, branches of neuroscience such as psychiatry, parapsychology, somnambulism, hypnosis and trance states were fields of the mind that persuaded and fascinated him, and he devoted time to their study despite how intangible these investigations turned out to be. During his time as a professor at the University of Valencia, he even created a Committee for Psychological Research, a dedication that he abandoned because it interfered his daily biological and teaching work, and even with his days off, reserved exclusively for country excursions. Even so, time later he wrote a book on *“hypnotism, spiritism, and metapsychics”* which he finally declined to edit (Ramón y Cajal, 1934, p. 218), but had published a pioneering article on the successful application of hypnosis as an anesthetic preparation for childbirth (Ramón y Cajal, 1889). Over time, he continued to be interested in certain mental manifestations in which voluntary behavior was transiently overridden, and in this sense the oneiric sphere through which he tried to glimpse the true psychoanalytic meaning of reverie is very singular (Ehrlich, 2017). Shortly before his death he wanted to compile a series of dreamlike experiences in an essay that he intended to call *“The hallucinations of reverie”* (Ramón y Cajal, 1934, p. 46), but of the more than half a 1,000 daydreams that he wrote down and analyzed, only a little more than a 100 have been preserved, almost all corresponding to his senescence, when the depressive anxiety came to not leave him. In those final years of his life he showed important internal conflicts, not being strange that with suspicious frequency his dreams manifested a regression to childhood or were related to scenes of Nature, the mountains, the sea or travels (Rallo et al., 2014). The spectrum of consciousness, the construction of thought and the relationship between mind and brain were aspects that also aroused the interest of two foreign colleagues who were very close to Cajal –Charles Sherrington (1857–1952) and Wilder Penfield (1891–1976)–, and despite being celebrated scientists for their anatomical and physiological discoveries of the nervous system, toward the end of their lives both conducted their respective broad and deep inquiries into the unfathomable field of the mental sphere (Sherrington, 1940; Penfield, 1975).

Despite all his forays into the enigmatic fields of mind and the fact that Cajal was a world-renowned neuroscientist, his incessant curiosity to find the construct of mental thought was, however, unsuccessful. In the following stanza, written almost at the age of 50, he recognizes this fact by manifesting ingenious symbolisms with Nature:

*“Seven lustrums have passed since then, and I still do not know where it is. And with each day the conviction that the world and life in its intimate springs are today unintelligible grows stronger in me. And I begin to think that the forgers of systems, the proud philosophers who aspire to definitively set the course of Humanity, are comparable to the madman who, on a gloomy night and standing on the top of Montblanc, solemnly lit a match and he will boast of lighting up the Universe with it!”* (Ramón y Cajal, 1901, pp. 251–252).

On the phylogenetic scale, Nature, despite its imperfections, had never created an organ as sublime as the human brain, an ingenuity so complex and exclusive that it was capable of generating the most absorbed and elevated thought. With the following words he begins his great book *Textura del sistema nervioso del hombre y de los vertebrados* [Texture of the nervous system of man and vertebrates]: *“The nervous system represents the last term in the evolution of living matter and the most complicated machine with the noblest activities that nature offers us”* (Ramón y Cajal, 1899a, p. 1). Over the years, he trusted that good fortune would provide eternity to that prodigious device, for whose glorious mission Nature herself had designed it. Let us observe how he philosophized about it in a beautiful way:

*“… the encephalon, instead of being a fickle balloon hovering in mist and the plaything of an ignored meteorology, will become a perfect and steerable aerostat, capable of following its destiny undaunted, insensitive to the onslaught of the wind and the threat of lightning… Then, the human being will truly be king of creation, because he will have achieved the most glorious and transcendental triumph of life: the conquest of his own brain; that is, the clarification of the formidable mystery; the solemn taking possession of the sacred ark, summary and synthesis of the cosmos, in whose bosom the germs of the eternal truths sleep inviolate”* (Ramón y Cajal, 1905c, pp. 20–21).

But, during his senescence, Cajal tried to appease his sad mood, because he succumbed to an abstruse duality, the one that confronts between the destruction of the highest matter ever created or its immortality after a perpetual fusion with the cosmic energy:

*“And what a disconsolate indifference of Nature to throw away like vile dross the masterpiece of Creation, the sublime cerebral mirror where it acquires self-consciousness!”* (Ramón y Cajal, 1923, p. 83).

*“From the summit of eternity, human heads must seem to the psychological principle of the Universe like those bubbles of foam produced in the wave as it breaks on the beach. They shine for a moment with polychromatic lights, copy in miniature the blue of the sky and the magic of the landscape and explode instantly, giving way to the new generation of iridescent globules”* (Ramón y Cajal, 1920, p. 53).

Feeling trapped during his last years under arcane thoughts and questions about death, Cajal had begun to write a book entitled *“Solos ante el misterio”* [Alone in front of the mystery] (Ramón y Cajal, 1934, p. 25), an esoteric essay on the afterlife, whose contents mysteriously disappeared without a trace –authors very close to Cajal referred to this book with the title *“El misterio ante la tumba”* [Mystery in front of the tomb] (Durán and Alonso, 1983, p. 436)−. It would have been really fascinating to know the transcendental oneiric meditation that the savant Cajal had in store for us in this manuscript, perhaps he made an analysis of the mental process experienced in the first moments after the end of a life. Probably in those pages he was also capturing a deep existential introspection under the voluptuousness of the soul, as ethereal and intangible essence, during the emotional trance in the last trip to enigmatic scenarios that, surely, would be flooded with allegories about a sublime idealized and dreamed Nature.

As Cajal grew older, in moments of melancholy he felt nostalgic for not having enjoyed enough of the natural environments so loved. The years were piling up on him, with their relentless way of weaving the incessant and uncertain becoming. He spent a lot of time confined in his neurohistological laboratory and dedicated to the University, and despite the exceptional scientific and academic teaching work already carried out, his life passed perhaps transporting him toward unfulfilled dreams. Some of his own testimonies seem to reveal to us his deep heartbeat and were evidence of some existential weaknesses that assaulted his lucid and multifaceted instinct. The union of science and art led Cajal to a happy existence, although, the random destiny spoiled him a more bohemian and calm life:

*“A limited horizon makes us miss the expanse of the mountains* […] *The discipline of study and the austere regime of the classroom are the cause that we covet the life of the outdoors and the freedom to arbitrarily choose the subject of our thoughts”* (Ramón y Cajal, 1901, p. 171).

*“As I have progressed in the narrative, my ‘autobiography’ has become ‘depersonalized’. Regular work and the spirit of adventure are incompatible things. From increasingly poorer in enjoyable episodes, my life has been gradually absorbed by my work. The bee has been forgotten in consideration of the honeycomb”* (Ramón y Cajal, 1917, p. 575).

It was not a coincidence that he expressly highlighted the words ‘autobiography’ and ‘depersonalized,’ and that he reserved this second meditation for the epilog of his book *Historia de mi labor científica* [History of my scientific work]. However, Cajal tried to combine his scientific work with evasion in the countryside and, whenever he could, he bathed his soul in attractive places surrounded by Nature. As if it were an urgent need, in natural environments could he develop his exquisite sensitivity and revitalize his creative activity. Like nowhere else, it was in those places full of pleasant sensations that allowed him to find complete peace and thus regain mental balance while his imagination wandered incessantly. In many moments of mental fatigue, of fruitless scientific work, even of boredom spent in solitude, Cajal turned to those healing places of the body and spirit that called him:

*“It is remarkable how at the first touch of the fresh and brave air, the gloomy ideas dissipate like fog before the sun: Little by little we feel penetrated by the august serenity of the heavens, which, passing from the eyes to the heart, drives away black melancholies and restores the joy of living”* (Ramón y Cajal, 1901, pp. 282–283).

These immersions in Nature would enlighten his mind a lot. The following testimonies about the mountainous surroundings of Madrid, the city where he lived during the last 42 years of his life, are very illustrative:

*“Lying in the meadows surrounding the city, in view of the snowy Guadarrama mountain range, I still like to give free rein to my thoughts today”* (Ramón y Cajal, 1901, p. 45); *“How many small discoveries are associated in my memory with such a solitary footpath in Moncloa, or with such an ash tree on the banks of the Manzanares, or with some hill in Amaniel or in the Dehesa de la Villa, splendid viewpoints from which the Guadarrama, leaning out among the pines, shows all its serene majesty!”* (Ramón y Cajal, 1917, p. 237).

Even when the years led him to deafness and arteriosclerosis, conditioning him to a certain hypochondriac and misanthropic state, the elder Cajal never ceased to delight in strolling alone through emblematic urban gardens, fleeing from the social glare. At that advanced stage of his life, he liked to frequent an extensive park that was close to his house, where inspirations for two of his most important scientific works were surely born, *Estudios sobre la degeneración y regeneración del sistema nervioso* [Studies on the degeneration and regeneration of the nervous system] (Ramón y Cajal, 1913, 1914) and what it is considered his scientific testament, entitled *¿Neuronismo o reticularismo?* [Neuronism or reticularism?] (Ramón y Cajal, 1933):

*“In the great park of the city of Madrid, the ‘Buen Retiro,’ along well swept paths between chestnut trees arranged in squares and quincunxes like an Arabian garden, the Professor took his favorite walk* […] *He pondered, he dreamed, but that did not prevent the enjoyable use of his senses. His eyes still looked brilliant, still saw much”* (Williams, 1954, p. 220).

**Figure 9:**
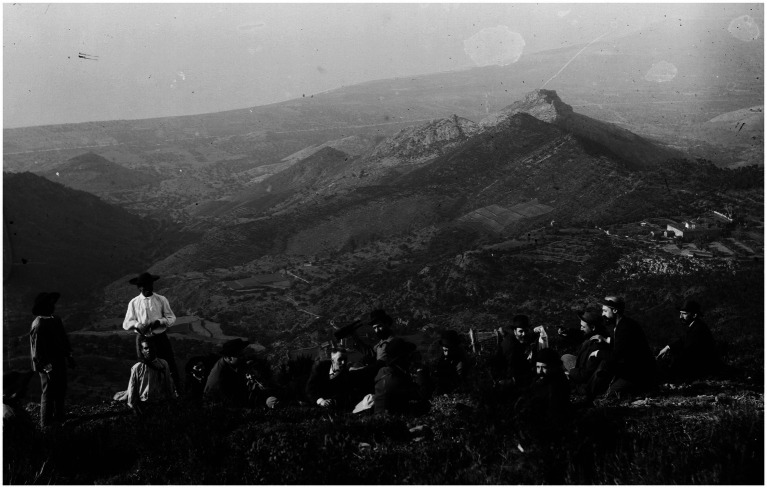
Cajal photographs a group of scientific colleagues with some rural farmers at the top of the Sierra Litoral in front of the coast of the Mediterranean Sea, in Benicasim, Spain (ca. 1886) [from Cajal Legacy-CSIC, Madrid, Spain].

**Figure 10:**
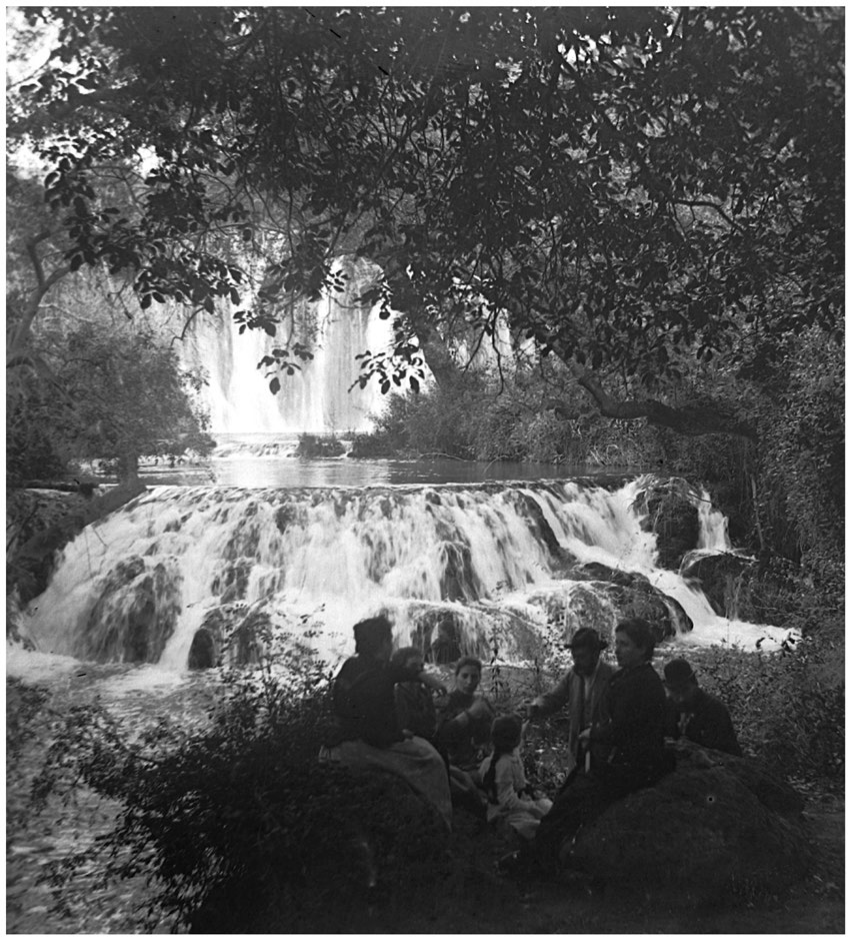
Cajal photographs some members of his beloved family during a tour through the forests and waterfalls of the Piedra River, in Nuévalos, Spain (ca. 1893) [from Cajal Legacy-CSIC, Madrid, Spain].

**Figure 11:**
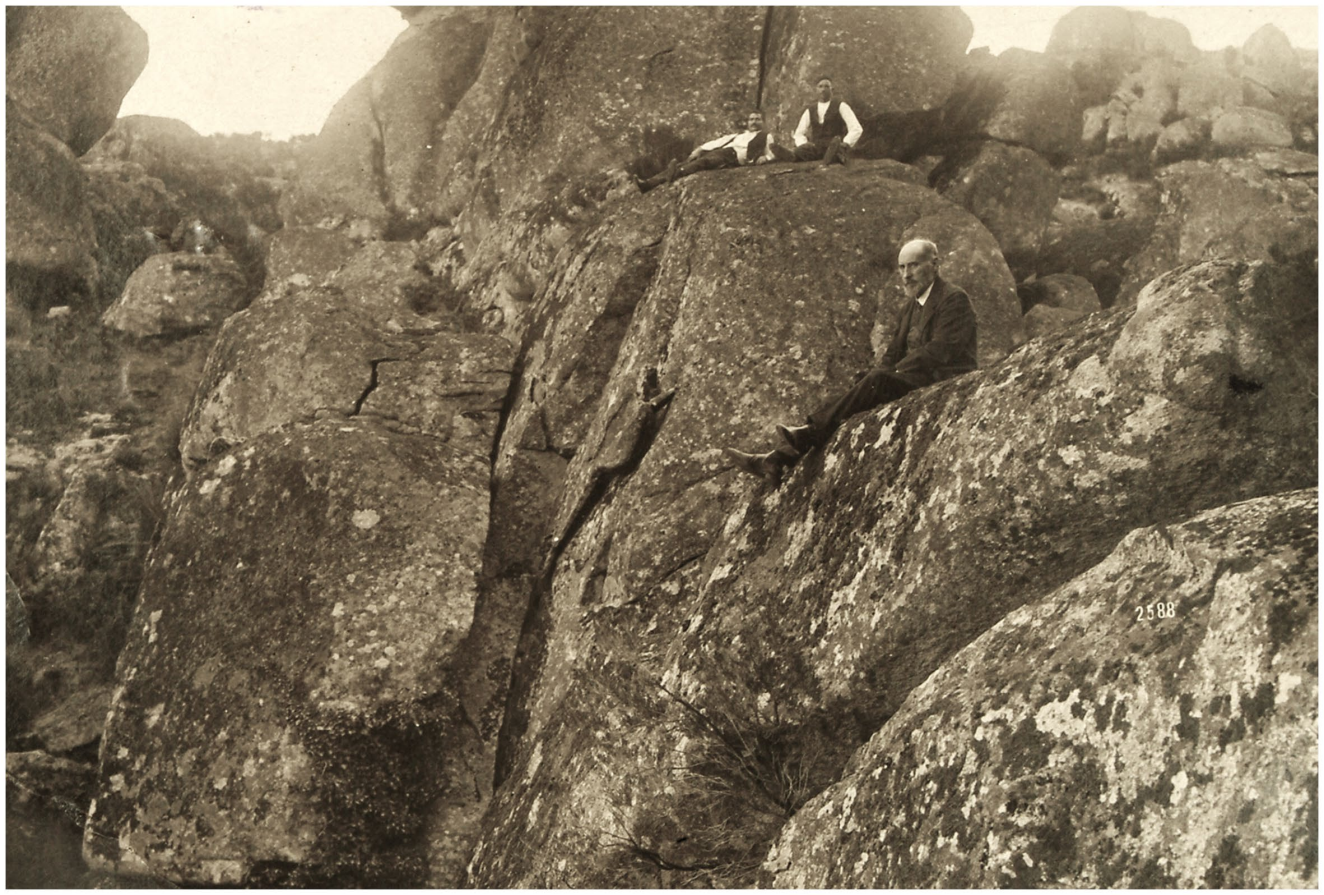
Ramón y Cajal portrayed with some guides and perched on the rocks during an excursion in the Cabreiroá mountains, in Galicia, Spain (y. 1909) [from Cajal Legacy-CSIC, Madrid, Spain].

**Figure 12:**
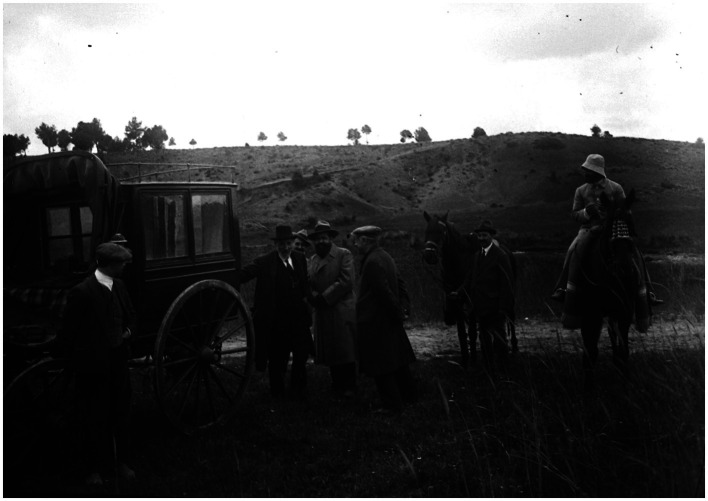
Santiago Ramón y Cajal in his old age with some comrades during a naturalistic excursion through the highlands of Cuenca, Spain (y. 1912) [from Cajal Legacy-CSIC, Madrid, Spain].

During his holidays or even when he retired from his professorship, Cajal continued to travel to different places, taking countryside and naturalist excursions, as well as undertaking some mountain ascents (Figures 9–12). He enjoyed many of the wonderful landscapes of Europe, but above all he was seduced by the lands and horizons of Spain, his beloved country. He especially liked to relax in idyllic mountain settings in the Pyrenees, the Sierra de Guadarrama or the Picos de Europa, and also on the attractive sea coasts of the Mediterranean and the Cantabrian. He was even able to enjoy all that varied Nature until very shortly before he died.

Cajal’s naturalistic inclination was such that it was not in vain that he wanted to publish some 20 of his studies and writings in the *Anales de la Sociedad Española de Historia Natural* [Annals of the Spanish Society of Natural History] and in the journal *La Naturaleza* [The Nature]. He also became an elected member of several Academies and Institutions of Natural Sciences, including president of the Royal Spanish Society of Natural History in 1897, and honorary president in 1932, positions of which he always felt especially proud and honored. In this context, it is worth mentioning that there were many colleagues from different fields with whom Cajal would share, throughout his life, their mutual love for Nature; however, focusing among his admired neuroscientists, some of whom belong to the Spanish neurological school, the following stand out: Domingo Sánchez (1860–1947), Spanish physician and naturalist who had lived years of scientific adventures in the tropical jungle of the Pacific Ocean and later collaborated closely for decades with Cajal since the two met in 1899; Sánchez contributed significantly to the histological and physiological knowledge of the nervous system of invertebrates (Serrano-Herrera and Espinosa-Sánchez, 2024). Fernando de Castro (1896–1967), Spanish physiologist and neuroanatomist, researcher of international prestige for carrying out the first description of a chemical and pressure biosensor (de Castro, 1926; Ros-Bernal and de Castro, 2020), and who maintained a loyal and long-standing friendship with Cajal since 1918, increased by their mutual love of mountaineering. The Italian Angelo Mosso (1846–1910), considered one of the fathers of Mountain Medicine for his daring studies in hypobaric hypoxia environment, among other works on the neurophysiology of high-altitude exposures in the Alps (Mosso, 1898); Cajal met him in Turin in 1889 and during the long trip they made to the United States of America at the invitation of Clark University in 1899. The British Charles Scott Sherrington (1857–1952), neurophysiologist awarded the Nobel Prize in 1932 and who had a deep fascination with Nature (Sherrington, 1940) since his early fondness for the mountains, as he was an outstanding pioneer of snow sports in the Swiss alpine region of Grindelwald; He and Cajal maintained a close friendship that lasted over the years since Cajal was hosted in his London home in 1894 (de Carlos and Molnár, 2020). The Norwegian Fridtjof Nansen (1861–1930), whose promising neuroscientific career (Nansen, 1887) was overshadowed by a brilliant life of oceanographic exploration voyages, as well as boreal incursions that had never been carried out before (Edwards and Huntford, 1998); although Cajal referred to Nansen on several occasions in his scientific and literary books –even defending with express forcefulness his forgotten important role in neurohistology–, and that in a hitherto unpublished letter that Cajal sent to his fellow Scandinavian colleague Gustav Retzius in 1898, he mentions *“intrepid and friendly Nansen”* (Fernández, 2014, p. 497), it is unknown what the meetings between the two really were.

Santiago Ramón y Cajal, the ‘father of modern neuroscience’, did not betray his adventurous desires of childhood and youth, because he never wanted to distance himself from that child who dreamed of conquering virgin landscapes and horizons:

*“What would be there among those peaks crowned with perpetual snow, still, solemn, silent and immutable…?* […] *Who knows if from the highest Pyrenean summit there will not appear crystalline and serene lakes like mirrors, bordered by towering cliffs and sharp and painted rocks, through which streams plunge in iridescent waterfalls…!* […] *That insane curiosity tormented me; and if my forces had been powerful enough to give summit to the desired enterprise, I would have thrown myself without hesitation on the colossal back of the giant, and I would have climbed the high peaks”* (Ramón y Cajal, 1901, pp. 116–117).

The vital essence that motivated the unfolding of his days and the passionately romantic and pure sense with which he directed his steps through life was aptly summed up by an old friend and childhood playmate along the wild fields and rivers of the land where Cajal grew up. Seduced by the implicit truth of such testimony, Cajal wanted to reproduce it somewhat synthesized in successive editions of the book about his own memories; but the original text of his medical colleague, published years earlier in the social press, concludes in a revealing way, given that in the most intimate texture of neuroanatomy Cajal’s existential circle seemed definitively closed:

*“… The distinguished histologist revived, as a child, the semi-legendary idea of nautical adventures. He continued to believe in his island. He sailed, got his bearings and arrived victoriously. The island existed! In the nerve centers, in the spinal cord and in the brain, the ‘Island of Cajal’ is indeed found”* (Salillas, 1901, p. 1).

## Conclusion

Cajal contributed with extreme delicacy and candor to the development of neuroscience. All his work, be it scientific or any other genre, is brimming with revelations of compelling sensitivity and sentimentality for Nature, whether through apologies, symbolism or allegories toward it, and this is a categorical fact in his all-outstanding texts and drawings. This was a faithful reflection of how he saw, understood and enjoyed Nature with exquisite fervor since his childhood. But, his intense adventurous desires of youth for dreamlike surroundings and the deep thoughts that he developed during his maturity, reveal to us a pressing emotionality that was not sufficiently satisfied by the serious illnesses that he suffered early on. Neuroscience awakened in him the extreme curiosity to delve into the powerful mysteries of the mind and, especially, the histology of the nervous system offered him the opportunity to immerse himself in sublime landscapes of infinite beauty, through which he would venture into hidden routes and give free rein to his incorrigible adventurous impulse and his passionate and poetic creative imagination. A scientific romanticism that is unlikely to return in the current context of technological development, an old sentimental idealism thanks to which the foundations of modern neuroscience were laid, has been left behind. Aside from the obvious pure and clear scientific motivations that Cajal showed to achieve a strong advance in science, his complex personality was always influenced by his determined persuasion toward Nature in all its manifestations, since this meant for him its existential essence, his vital axis and inexhaustible source of consolation and inspiration where he poured all his fantasy. The desire of a vocational adventurer, of an artist and a humanist, of an eminent sentimental soul who knew how to link in a wonderful way, as few have done, Neuroscience and Nature.

## Author contributions

EG: Conceptualization, Investigation, Writing – original draft, Writing – review & editing.

## References

[ref1] Albarracín A. (1997). Ramón y Cajal. Explorador de selvas vírgenes. An. Sem. His. Filos. 14, 171–183.

[ref2] Amunts K. Ebell C. Muller J. Telefont M. Knoll A. Lippert T. (2016). The Human Brain Project: creating a European research infrastructure to decode the human brain. Neuron 92, 574–581. doi: 10.1016/j.neuron.2016.10.046, PMID: 27809997

[ref3] de Carlos J. A. Molnár Z. (2020). Cajal’s interactions with Sherrington and the Croonian lecture. Anat. Rec. 303, 1181–1188. doi: 10.1002/ar.24189, PMID: 31172626

[ref4] de Carlos J. A. Pedraza M. (2014). Santiago Ramón y Cajal: the Cajal Institute and the Spanish histological school. Anat. Rec. 297, 1785–1802. doi: 10.1002/ar.23019 25125377

[ref5] de Castro F. (1926). Sur la structure et l’innervation de la glandeinter-carotidienne (glomus caroticum) de l’homme et desmammifères, et sur un nouveau système d’innervation autonomedu nerf glosopharyngien. Trav. Lab. Rech. Biol. 24, 365–432.

[ref6] de Castro F. (2019). Cajal and the Spanish neurological school: neuroscience would have been a different story without them. Front. Cell. Neurosci. 13:187. doi: 10.3389/fncel.2019.00187 31178695 PMC6542961

[ref7] de Castro F. (2021). El arte que alumbró la moderna neurociencia: El dibujo científico de Cajal y sus discípulos. Kranion. 16, 146–158. doi: 10.24875/kranion.m21000017

[ref8] DeFelipe J. (2006). Brain plasticity and mental processes: Cajal again. Nat. Rev. Neurosci. 7, 811–817. doi: 10.1038/nrn2005, PMID: 16988656

[ref9] DeFelipe J. (2018). Cajal’s Neuronal Forest: Science and Art. New York: Oxford University Press.

[ref10] DeFelipe J. Garrido E. Markram H. (2014). The death of Cajal and the end of scientific romanticism and individualism. Trends Neurosci. 37, 525–527. doi: 10.1016/j.tins.2014.08.002, PMID: 25288199

[ref11] Durán G. Alonso F. (1983). Cajal, Escritos Inéditos. Barcelona: Editorial Científico Médica.

[ref12] Edwards S. Huntford R. (1998). Fridtjof Nansen: from the neuron to the North Polar Sea. Endeavour 22, 76–80. doi: 10.1016/S0160-9327(98)01118-1, PMID: 9719772

[ref13] Ehrlich B. (2017). The Dreams of Santiago Ramón y Cajal. New York: Oxford University Press.

[ref14] Fernández J. A. (2014). Ramón y Cajal: Epistolario. Madrid: La Esfera de los Libros.

[ref15] Ferreira F. R. M. Nogueira M. DeFelipe J. (2014). The influence of James and Darwin on Cajal and his research into the neuron theory and evolution of the nervous system. Front. Neuroanat. 8:1. doi: 10.3389/fnana.2014.00001, PMID: 24523676 PMC3905238

[ref16] García-López P. García-Marín V. Freire M. (2010). The histological slides and drawings of Cajal. Front. Neuroanat. 4:9. doi: 10.3389/neuro.05.009.2010, PMID: 20339483 PMC2845060

[ref17] Garrido E. (2014). Nature, mountains, sport and adventure in the life of Santiago Ramón y Cajal. Cultura Ciencia Deporte. 9, 69–80. doi: 10.12800/ccd.v9i25.390

[ref18] Garrido E. (2016). Cajal y la Naturaleza. Vivencias y Pensamientos. Madrid: Ed. Desnivel.

[ref19] Giné E. Martínez C. Sanz C. Nombela C. de Castro F. (2019). The women neuroscientists in the Cajal school. Front. Neuroanat. 13:72. doi: 10.3389/fnana.2019.00072 31379519 PMC6646472

[ref20] Goering S. Yuste R. (2016). On the necessity of ethical guidelines for novel neurotechnologies. Cell 167, 882–885. doi: 10.1016/j.cell.2016.10.029, PMID: 27814514

[ref21] Hadzigeorgiuo Y. Schulz R. (2014). Romanticism and romantic science: their contribution to science education. Sci. Educ. 23, 1963–2006. doi: 10.1007/s11191-014-9711-0

[ref22] Ibarz V. (1994). La Psicología en la Obra de Santiago Ramón y Cajal. Zaragoza: Institución Fernando el Católico.

[ref23] Jacobson M. (1993). Foundations of Neuroscience. New York: Springer Science-Business Media, LLC.

[ref24] Lewy E. (1987). Santiago Ramón y Cajal: el Hombre, el Sabio y el Pensador. Madrid: Extensión Científica y Acción Cultural del CSIC.

[ref25] Mosso A. (1898). Life of Man on the High Alps. London: T. Fisher Unwin.

[ref26] Nansen F. (1887). The Structure and Combination of the Histological Elements of the Central Nervous System. Bergens museums Aarsberetning for. Bergen: John Grieg.

[ref27] Newman E. A. Araque A. Dubinsky J. M. Swanson L. W. King L. Himmel E. (2017). The Beautiful Brain. The Drawings of Santiago Ramón y Cajal. New York: Abrams.

[ref28] Olóriz F. (1907). Discursos leídos ante la Real Academia de Medicina en la recepción pública de don S. Ramón y Cajal. [dissertation]. [Madrid]: Ambrosio Pérez y Cª, 55–84.

[ref29] Penfield W. (1975). The Mystery of the Mind: a Critical Study of Consciousness and the Human Brain. Princeton: Princeton University Press.

[ref30] Rallo J. Martí F. Jiménez-Arriero M. A. (2014). Los Sueños de Santiago Ramón y Cajal. Sus Teorías sobre el Ensueño desde la Crítica a las Teorías Oníricas de Freud. Madrid: Editorial Biblioteca Nueva S.L.

[ref31] Ramón y Cajal S. (1889). Dolores del parto considerablemente atenuados por la sugestión hipnótica. Gaceta Médica Catalana. 12, 484–486.

[ref32] Ramón y Cajal S. (1891). Pequeñas Contribuciones al Conocimiento del Sistema Nervioso. Barcelona: Imprenta de la Casa Provincial de Caridad.

[ref33] Ramón y Cajal S. (1893). Nuevo Concepto de la Histología de los Centros Nerviosos. Barcelona: Imprenta de Henrich y Cª, Sucesores de N. Ramírez.

[ref34] Ramón y Cajal S. (1894a). The Croonian Lecture: La fine structure des centres nerveux. Proc. R. Soc. Lond. 55, 444–468.

[ref35] Ramón y Cajal S. (1894b). Consideraciones Generales sobre la Morfología de la Célula Nerviosa. Madrid: Imprenta de Nicolás Moya.

[ref36] Ramón y Cajal S. (1897). Fundamentos racionales y condiciones técnicas de la investigación biológica. [dissertation]. [Madrid]: Imprenta de L. Aguado, 5–82.

[ref37] Ramón y Cajal S. (1899a). Textura del Sistema Nervioso del Hombre y de los Vertebrados (Tomo I). Madrid: Imprenta y Librería de Nicolás Moya.

[ref38] Ramón y Cajal S. (1899b). Reglas y Consejos sobre Investigación Biológica. Madrid: Imprenta de Fortanet.

[ref39] Ramón y Cajal S. (1901). Recuerdos de mi Vida: Mi Infancia y Juventud. Madrid: Imprenta de Fortanet.

[ref40] Ramón y Cajal S. (1904). Textura del Sistema Nervioso del Hombre y de los Vertebrados (Tomo II). Madrid: Imprenta y Librería de Nicolás Moya.

[ref41] Ramón y Cajal S. (1905a). Discurso en homenaje a Echegaray. Madrid Científico 481, 127–128.

[ref42] Ramón y Cajal S. (1905b). Cuentos de Vacaciones (Narraciones Pseudocientíficas). Madrid: Imprenta de Fortanet.

[ref43] Ramón y Cajal S. (1905c). “Prólogo” in Introducción al Estudio de la Psicología Positiva. ed. Maestre T. (Madrid: Librería Editorial de Bailly-Bailliere e Hijos), 10–21.

[ref44] Ramón y Cajal S. (1913). Estudios sobre la Degeneración y Regeneración del Sistema Nervioso (Tomo I). Madrid: Imprenta de Hijos de Nicolás Moya.

[ref45] Ramón y Cajal S. (1914). Estudios sobre la Degeneración y Regeneración del Sistema Nervioso (Tomo II). Madrid: Imprenta de Hijos de Nicolás Moya.

[ref46] Ramón y Cajal S. (1917). Recuerdos de mi Vida: Historia de mi Labor Científica. Madrid: Imprenta y Librería de Nicolás Moya.

[ref47] Ramón y Cajal S. (1920). Chácharas de Café: Pensamientos, Anécdotas y Confidencias. Madrid: Imprenta y Librería de Nicolás Moya.

[ref48] Ramón y Cajal S. (1921). Las sensaciones de las hormigas. Arch. Neurobiol. 2, 1–21.

[ref49] Ramón y Cajal S. (1923). Recuerdos de mi Vida. Madrid: Imprenta de Juan Pueyo.

[ref50] Ramón y Cajal S. (1924). Trabajos Escogidos (1880–1890). Madrid: Publicaciones de la Junta para el homenaje a Cajal.

[ref51] Ramón y Cajal S. (1933). ¿Neuronismo o reticularismo? Las pruebas objetivas de la unidad anatómica de las células nerviosas. Arch. Neurobiol. 13, 1–144.

[ref52] Ramón y Cajal S. (1934). El Mundo Visto a los Ochenta Años: Impresiones de un Arteriosclerótico. Madrid: Tipografía Artística.

[ref53] Ros-Bernal F. de Castro F. (2020). Fernando de Castro: Cajal’s man on the peripheral nervous system. Anat. Rec. 303, 1206–1214. doi: 10.1002/ar.24191 31172650

[ref54] Rose N. (2014). The Human Brain Project: social and ethical challenges. Neuron 82, 1212–1215. doi: 10.1016/j.neuron.2014.06.001, PMID: 24945767

[ref55] Salillas R. (1901). La isla de Cajal. El Liberal. n° 8.073, 1. Madrid: Hemeroteca de la Biblioteca Nacional de España.

[ref56] Saltz J. (2018). Santiago Ramón y Cajal, a Nobel laureate in medicine, deserves a play next to Michelangelo and Leonardo as a draftsman. New York Magazine. New York: Vox Media Network. Available at: https://www.vulture.com/2018/03/the-doctor-whose-drawings-rival-michelangelos.html

[ref57] Serrano-Herrera A. Espinosa-Sánchez J. M. (2024). Domingo Sánchez y Sánchez (1860–1947): Cajal’s man on the nervous system of invertebrates. Front. Neuroanat. 17:1330452. doi: 10.3389/fnana.2023.1330452 38264082 PMC10803473

[ref58] Sherrington C. S. (1940). Man on his Nature. Cambridge: Cambridge University Press.

[ref59] Sherrington C. S. (1949). “Memoir of Dr. Cajal” in Explorer of the Human Brain: the Life of Santiago Ramón y Cajal (1852-1934). ed. Cannon D. F. (New York: Henry Schuman), 9–15.

[ref60] Steininger H. Codina-Canet C. (2018). Santiago Ramón y Cajal – neuroanatomist, artist and writer. Dtsch. Med. Wochenschr. 143, 1847–1851. doi: 10.1055/a-0640-4652, PMID: 30562819

[ref61] Verweyen J. M. (2018). Naturphilosophie. London: Forgotten Books.

[ref62] Williams H. (1954). Don Quixote of the Microscope: an Interpretation of the Spanish Savant Santiago Ramón y Cajal (1852-1934). London: Jonathan Cape.

[ref63] Yuste R. (2015). The discovery of dendritic spines by Cajal. Front. Neuroanat. 9:18. doi: 10.3389/fnana.2015.00018, PMID: 25954162 PMC4404913

[ref64] Yuste R. Bargmann C. (2017). Toward a global BRAIN initiative. Cell 168, 956–959. doi: 10.1016/j.cell.2017.02.023, PMID: 28256259

